# (6′R*,7′R*)-7′-(1,3,-Diphenyl-1*H*-pyrazol-4-yl)-1,2,5′,6′,7′,7a’,3′′,4′′-octa­hydro-1′*H*,2′′*H*-dispiro­[acenaphthyl­ene-1,5′-pyrrolo­[1,2-*c*][1,3]thia­zole-6′,3′′-[1]benzopyran]-2,4′′-dione

**DOI:** 10.1107/S1600536813005825

**Published:** 2013-03-06

**Authors:** J. Murugan, J. Haribabu, B. S. R. Reddy, G. Rajarajan, S. Murugavel

**Affiliations:** a Department of Physics, Sree Krishna College of Engineering, Anicut, Vellore 632 001, India; b Industrial Chemistry Labratory, Central Leather Research Institute, Adyar, Chennai 600 020, India; c Department of Physics, Mahendra Engineering College, Namakkal 637 503, India; d Department of Physics, Thanthai Periyar Government Institute of Technology, Vellore 632 002, India

## Abstract

In the title compound, C_40_H_29_N_3_O_3_S, the pyran ring adopts a sofa conformation, the thia­zolidine ring adopts a twisted conformation and the pyrrolidine ring adopts an envelope conformation with the N atom as the flap. The pyrazole ring is essentially planar [maximum deviation = 0.002 (2) Å] and forms dihedral angles of 4.8 (1) and 39.0 (1)°, respectively, with the benzene rings attached to the N and C atoms. The acenapthylene ring system is approximately planar [maximum deviation = 0.058 (2) Å] and forms dihedral angles of 85.9 (1) and 48.5 (1)°, respectively, with the pyrollothia­zole and chromene ring systems. The mol­ecular conformation is stabilized by three weak intra­molecular C—H⋯O hydrogen bonds, which generate one *S*(8) and two *S*(6) ring motifs. In the crystal, pairs of C—H⋯O hydrogen bonds link centrosymmetrically related mol­ecules into dimers, generating *R*_2_^2^(14) ring motifs. The crystal packing also features pairs of C—H⋯π inter­actions, which link the dimers into a supra­molecular chain along the *b* axis.

## Related literature

For the biological properties of spiro­heterocycles, see: Kilonda *et al.* (1995 ▶); Ferguson *et al.* (2005 ▶). For ring puckering parameters, see: Cremer & Pople (1975 ▶), and for asymmetry parameters, see: Duax *et al.* (1976 ▶). For related structures, see: Wei *et al.* (2012 ▶); Jagadeesan *et al.* (2013 ▶). For hydrogen-bond motifs, see: Bernstein *et al.* (1995 ▶).
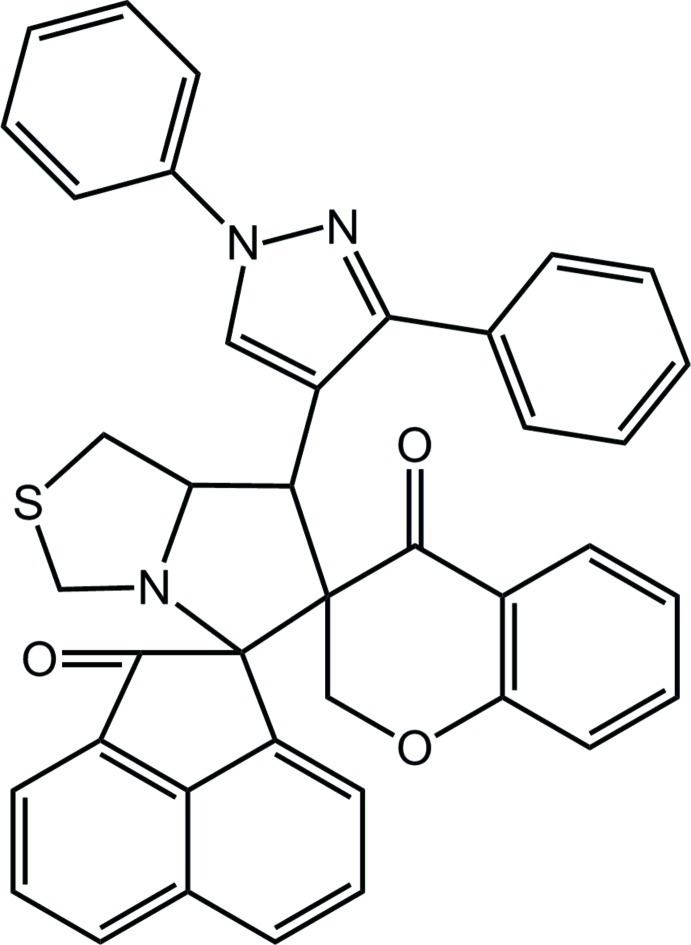


## Experimental

### 

#### Crystal data

C_40_H_29_N_3_O_3_S
*M* *_r_* = 631.72
Triclinic, 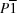

*a* = 9.9924 (6) Å
*b* = 13.2317 (8) Å
*c* = 13.2867 (8) Å
α = 116.900 (3)°
β = 92.325 (2)°
γ = 98.518 (3)°
*V* = 1537.79 (16) Å^3^
*Z* = 2
Mo *K*α radiation
μ = 0.15 mm^−1^
*T* = 293 K
0.23 × 0.21 × 0.16 mm

#### Data collection

Bruker APEXII CCD diffractometer
Absorption correction: multi-scan (*SADABS*; Sheldrick, 1996 ▶) *T*_min_ = 0.966, *T*_max_ = 0.976
36242 measured reflections
9612 independent reflections
6569 reflections with *I* > 2σ(*I*)
*R*_int_ = 0.029

#### Refinement

*R*[*F* ^2^ > 2σ(*F* ^2^)] = 0.049
*wR*(*F* ^2^) = 0.154
*S* = 1.03
9612 reflections
424 parameters
H-atom parameters constrained
Δρ_max_ = 0.35 e Å^−3^
Δρ_min_ = −0.23 e Å^−3^

### 

Data collection: *APEX2* (Bruker, 2004 ▶); cell refinement: *APEX2* and *SAINT* (Bruker, 2004 ▶); data reduction: *SAINT* and *XPREP* (Bruker, 2004 ▶); program(s) used to solve structure: *SHELXS97* (Sheldrick, 2008 ▶); program(s) used to refine structure: *SHELXL97* (Sheldrick, 2008 ▶); molecular graphics: *ORTEP-3 for Windows* (Farrugia, 2012 ▶); software used to prepare material for publication: *SHELXL97* and *PLATON* (Spek, 2009 ▶).

## Supplementary Material

Crystal structure: contains datablock(s) global, I. DOI: 10.1107/S1600536813005825/tk5201sup1.cif

Structure factors: contains datablock(s) I. DOI: 10.1107/S1600536813005825/tk5201Isup2.hkl

Additional supplementary materials:  crystallographic information; 3D view; checkCIF report

## Figures and Tables

**Table 1:** Hydrogen-bond geometry (Å, °) *Cg* is the centroid of the C23–C28 benzene ring.

*D*—H⋯*A*	*D*—H	H⋯*A*	*D*⋯*A*	*D*—H⋯*A*
C7—H7⋯O3	0.93	2.47	3.207 (2)	136
C29—H29*A*⋯O3	0.97	2.45	3.074 (2)	122
C17—H17⋯O3	0.98	2.52	3.091 (2)	117
C38—H38⋯O1^i^	0.93	2.40	3.226 (2)	148
C1—H1⋯*Cg* ^ii^	0.93	2.94	3.713 (3)	142

Symmetry codes: (i) 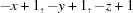; (ii) 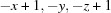.

**Fig. 1.**
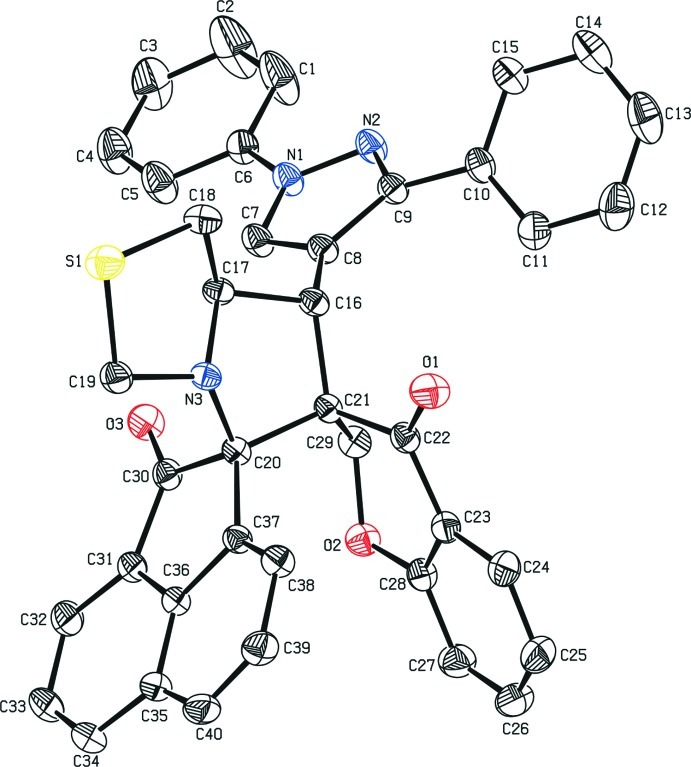
The molecular structure of the title compound with the atom numbering scheme. Displacement ellipsoids are drawn at the 30% probability level. H atoms are omitted for the sake of clarity.

**Fig. 2.**
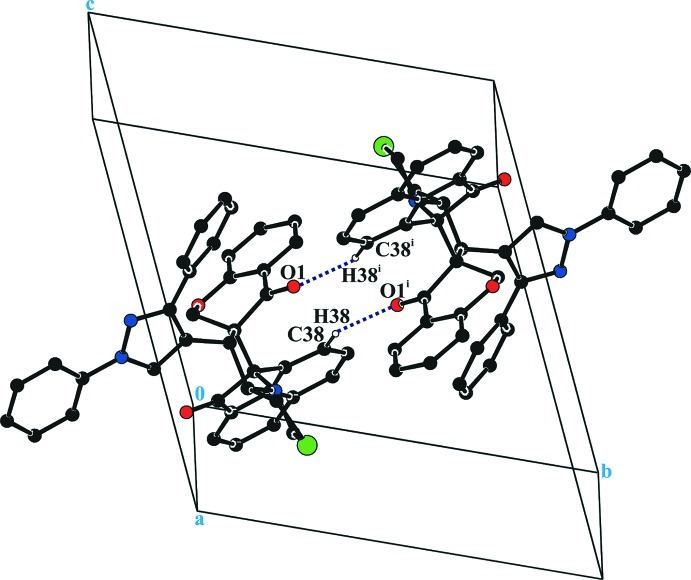
Part of the crystal structure of the title compound showing C—H···O intermolecular hydrogen bonds (dotted lines) generating an R22(14) centrosymmetric dimer [Symmetry code: (i) 1-x, 1-y, 1-z].

**Fig. 3.**
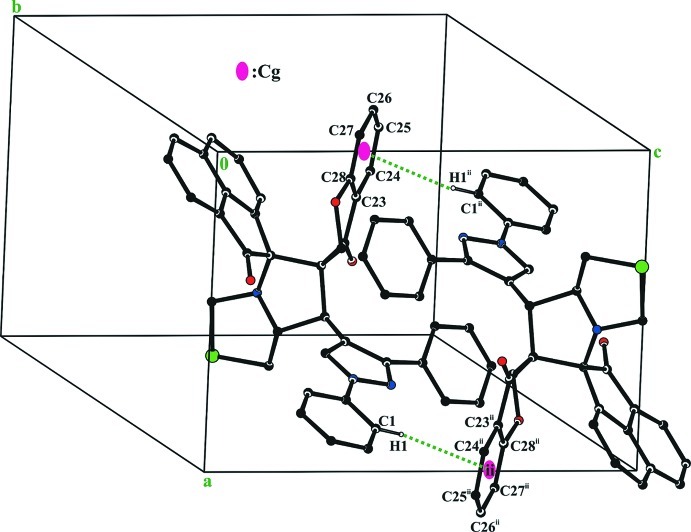
Part of the crystal structure of (I), showing the formation of a cyclic centrosymmetric dimer. For the sake of clarity, H atoms not involved in the motif shown have been omitted. Cg denotes centroid of the C23–C28 benzene ring. [Symmetry code: (ii) 1-x, -y, 1-z].

## supplementary crystallographic information

### Comment

The design and synthesis of glycospiroheterocycles attracts interest because of the synthetic challenges they present and their biological profile against viruses, bacteria and cancer cells (Ferguson *et al.*, 2005). Pyrrolidines, pyrroles and chromenes are common structural motifs in drugs and drug candidates owing to their ability to act as selective glycosidase inhibitors, which are used in the treatment of diabetes, cancer, malaria and viral infections, including AIDS (Kilonda *et al.*, 1995). In view of this biological importance, the crystal structure of the title compound has been determined and the results are presented here.

Fig. 1. shows a displacement ellipsoid plot of (I), with the atom numbering scheme. The pyran ring (O2/C21/C22/C23/C28/C29) adopts a sofa conformation with puckering parameters (Cremer & Pople, 1975), Q_T_ = 0.499 (2) Å, θ = 127.1 (2)°, φ = 102.3 (2)°. The thiazolidine (S1,N3,C17–C19) ring adopts a twist conformation, with twist about the C17—N3 bond; the puckering parameters q_2_ = 0.473 (2) Å and φ_2_ = 267.1 (2)°, and asymmetry parameter (Duax *et al.*, 1976) ΔC_2_[C17—N3] = 2.0 (1) Å. The pyrrolidine (N3/C16/C17/C20/C21) ring adopts an envelope conformation with the N3 (displacement = 0.273 (1) Å) atom as the flap atom and with puckering parameters q_2_ = 0.4062 (2) Å and φ_2_ = 357.2 (2)°. The pyrazole ring (N1/N2/C7–C9) is essentially planar [maximum deviation = 0.002 (2) Å for atom C8] and the N- and C-bound benzene rings are inclined to this plane [dihedral angles = 4.8 (1) and 39.0 (1)°, respectively] and form a dihedral angle of 34.6 (1)° with each other. The acenapthalene ring system is approximately planar [maximum deviation = -0.058 (2) Å for atom C37] and forms dihedral angles of 85.9 (1)° and 48.5 (1)°, respectively, with the pyrollothiazole and chromene ring systems. The geometric parameters of the title molecule agrees well with those reported for similar structures (Wei *et al.*, 2012; Jagadeesan *et al.*, 2013).

The molecular structure is stabilized by a C7—H7···O3 intramolecular hydrogen bond, forming *S*(8) ring motif as well as intramolecular C29—H29A···O3 and C17—H17···O3 hydrogen bonds, both forming *S*(6) ring motifs (Bernstein *et al.*, 1995) (Table 1). In the crystal packing (Fig. 2), the centrosymmetrically related molecules are linked by C38—H38···O1 hydrogen bonds into cyclic centrosymmetric *R_2_^2^*(14) dimers. The crystal packing (Fig. 3) is further stabilized by C—H···π(arene) hydrogen bonds, in which atom C1 acts as a hydrogen bond donor *via* H1, to the C23–C28 benzene ring of a neighbouring molecule (symmetry operation: 1-*x*, -*y*, 1-*z*), thereby generating a cyclic centrosymmetric dimer (Table 1).

### Experimental

A mixture of Acenaphthoquinone (1.0 mmol), thioproline (1.1 mmol) and (*E*)-2,3-dihydro-3-((1,3-diphenyl-1H-pyrazol-4-yl) methylene)chromen-4-one (1.0 mmol) in ethanol was refluxed for 4h and cooled to room temperature. The solid formed in the reaction mixture was poured into water and filtered, dried, and recrystallized from ethanol to obtain the title compound in good yield (84–91%).

### Refinement

All the H atoms were positioned geometrically with C–H = 0.93–0.98 Å and constrained to ride on their parent atom, with *U*_iso_(H) =1.5*U*_eq_ for methyl H atoms and 1.2*U*_eq_(C) for other H atoms.

### Crystal data

**Table:**

C_40_H_29_N_3_O_3_S	*Z* = 2
*M**_r_* = 631.72	*F*(000) = 660
Triclinic, *P*1	*D*_x_ = 1.364 Mg m^−^^3^
Hall symbol: -P 1	Mo *K*α radiation, λ = 0.71073 Å
*a* = 9.9924 (6) Å	Cell parameters from 9836 reflections
*b* = 13.2317 (8) Å	θ = 1.8–31.0°
*c* = 13.2867 (8) Å	µ = 0.15 mm^−^^1^
α = 116.900 (3)°	*T* = 293 K
β = 92.325 (2)°	Block, colourless
γ = 98.518 (3)°	0.23 × 0.21 × 0.16 mm
*V* = 1537.79 (16) Å^3^	

### Data collection

**Table:**

Bruker APEXII CCD diffractometer	9612 independent reflections
Radiation source: fine-focus sealed tube	6569 reflections with *I* > 2σ(*I*)
Graphite monochromator	*R*_int_ = 0.029
Detector resolution: 10.0 pixels mm^-1^	θ_max_ = 31.0°, θ_min_ = 1.8°
ω scans	*h* = −14→14
Absorption correction: multi-scan (*SADABS*; Sheldrick, 1996)	*k* = −18→19
*T*_min_ = 0.966, *T*_max_ = 0.976	*l* = −19→17
36242 measured reflections	

### Refinement

**Table:**

Refinement on *F*^2^	Primary atom site location: structure-invariant direct methods
Least-squares matrix: full	Secondary atom site location: difference Fourier map
*R*[*F*^2^ > 2σ(*F*^2^)] = 0.049	Hydrogen site location: inferred from neighbouring sites
*wR*(*F*^2^) = 0.154	H-atom parameters constrained
*S* = 1.03	*w* = 1/[σ^2^(*F*_o_^2^) + (0.0749*P*)^2^ + 0.3833*P*] where *P* = (*F*_o_^2^ + 2*F*_c_^2^)/3
9612 reflections	(Δ/σ)_max_ < 0.001
424 parameters	Δρ_max_ = 0.35 e Å^−^^3^
0 restraints	Δρ_min_ = −0.23 e Å^−^^3^

### Special details

**Table:**

Geometry. All esds (except the esd in the dihedral angle between two l.s. planes) are estimated using the full covariance matrix. The cell esds are taken into account individually in the estimation of esds in distances, angles and torsion angles; correlations between esds in cell parameters are only used when they are defined by crystal symmetry. An approximate (isotropic) treatment of cell esds is used for estimating esds involving l.s. planes.
Refinement. Refinement of F^2^ against ALL reflections. The weighted R-factor wR and goodness of fit S are based on F^2^, conventional R-factors R are based on F, with F set to zero for negative F^2^. The threshold expression of F^2^ > 2sigma(F^2^) is used only for calculating R-factors(gt) etc. and is not relevant to the choice of reflections for refinement. R-factors based on F^2^ are statistically about twice as large as those based on F, and R- factors based on ALL data will be even larger.

### Fractional atomic coordinates and isotropic or equivalent isotropic displacement parameters (Å^2^)

**Table:**

	*x*	*y*	*z*	*U*_iso_*/*U*_eq_	
C1	0.7455 (3)	−0.28167 (19)	0.25650 (18)	0.0819 (8)	
H1	0.7796	−0.2454	0.3331	0.098*	
C2	0.7608 (4)	−0.3935 (2)	0.1866 (2)	0.1099 (13)	
H2	0.8045	−0.4326	0.2171	0.132*	
C3	0.7129 (3)	−0.44716 (19)	0.07414 (19)	0.0747 (7)	
H3	0.7228	−0.5228	0.0282	0.090*	
C4	0.6509 (2)	−0.38972 (17)	0.02968 (17)	0.0604 (5)	
H4	0.6194	−0.4254	−0.0475	0.073*	
C5	0.6343 (2)	−0.27878 (16)	0.09814 (16)	0.0555 (5)	
H5	0.5917	−0.2397	0.0669	0.067*	
C6	0.67997 (16)	−0.22511 (13)	0.21208 (13)	0.0384 (3)	
C7	0.61357 (16)	−0.03814 (13)	0.25043 (13)	0.0381 (3)	
H7	0.5798	−0.0559	0.1769	0.046*	
C8	0.62107 (14)	0.06520 (12)	0.34519 (12)	0.0333 (3)	
C9	0.67850 (15)	0.04757 (12)	0.43425 (13)	0.0344 (3)	
C10	0.71557 (16)	0.12760 (13)	0.55610 (13)	0.0375 (3)	
C11	0.63355 (19)	0.20395 (16)	0.61809 (15)	0.0480 (4)	
H11	0.5529	0.2062	0.5823	0.058*	
C12	0.6707 (3)	0.27680 (19)	0.73271 (18)	0.0653 (6)	
H12	0.6154	0.3283	0.7736	0.078*	
C13	0.7883 (3)	0.2736 (2)	0.78628 (18)	0.0743 (7)	
H13	0.8125	0.3224	0.8636	0.089*	
C14	0.8713 (2)	0.1981 (2)	0.72601 (18)	0.0662 (6)	
H14	0.9517	0.1964	0.7625	0.079*	
C15	0.83462 (19)	0.12508 (16)	0.61162 (15)	0.0484 (4)	
H15	0.8902	0.0737	0.5713	0.058*	
C16	0.59016 (14)	0.17413 (12)	0.34794 (12)	0.0313 (3)	
H16	0.6368	0.2390	0.4192	0.038*	
C17	0.64407 (14)	0.19223 (12)	0.24940 (12)	0.0318 (3)	
H17	0.6380	0.1176	0.1819	0.038*	
C18	0.78268 (15)	0.26687 (14)	0.26813 (15)	0.0417 (3)	
H18A	0.8548	0.2226	0.2576	0.050*	
H18B	0.7996	0.3319	0.3439	0.050*	
C19	0.58590 (16)	0.27596 (14)	0.13448 (14)	0.0397 (3)	
H19A	0.5457	0.3379	0.1335	0.048*	
H19B	0.5561	0.2066	0.0630	0.048*	
C20	0.41210 (13)	0.20580 (11)	0.23217 (11)	0.0293 (3)	
C21	0.43588 (13)	0.18533 (11)	0.33985 (11)	0.0292 (3)	
C22	0.41078 (15)	0.29222 (12)	0.44361 (12)	0.0336 (3)	
C23	0.26783 (15)	0.29257 (13)	0.46627 (13)	0.0369 (3)	
C24	0.22855 (18)	0.39297 (16)	0.54484 (15)	0.0468 (4)	
H24	0.2941	0.4591	0.5867	0.056*	
C25	0.0938 (2)	0.39515 (19)	0.56108 (17)	0.0552 (5)	
H25	0.0679	0.4626	0.6130	0.066*	
C26	−0.00309 (19)	0.29610 (19)	0.49947 (18)	0.0568 (5)	
H26	−0.0941	0.2974	0.5111	0.068*	
C27	0.03255 (17)	0.19627 (17)	0.42169 (17)	0.0497 (4)	
H27	−0.0336	0.1302	0.3811	0.060*	
C28	0.16822 (16)	0.19455 (14)	0.40403 (13)	0.0379 (3)	
C29	0.33824 (15)	0.08335 (13)	0.33603 (13)	0.0359 (3)	
H29A	0.3530	0.0127	0.2729	0.043*	
H29B	0.3570	0.0782	0.4056	0.043*	
C30	0.35466 (15)	0.08938 (12)	0.12151 (12)	0.0337 (3)	
C31	0.23951 (15)	0.10870 (13)	0.06378 (12)	0.0359 (3)	
C32	0.15924 (17)	0.04013 (16)	−0.03783 (14)	0.0480 (4)	
H32	0.1705	−0.0352	−0.0832	0.058*	
C33	0.05936 (18)	0.0870 (2)	−0.07148 (16)	0.0577 (5)	
H33	0.0040	0.0415	−0.1402	0.069*	
C34	0.04146 (18)	0.19750 (19)	−0.00610 (16)	0.0544 (5)	
H34	−0.0259	0.2252	−0.0313	0.065*	
C35	0.12287 (15)	0.27082 (16)	0.09887 (14)	0.0420 (3)	
C36	0.22067 (14)	0.22160 (13)	0.13097 (12)	0.0333 (3)	
C37	0.31473 (14)	0.28286 (12)	0.22961 (12)	0.0312 (3)	
C38	0.31458 (16)	0.39697 (13)	0.29630 (14)	0.0383 (3)	
H38	0.3788	0.4407	0.3600	0.046*	
C39	0.21453 (18)	0.44768 (15)	0.26665 (16)	0.0457 (4)	
H39	0.2123	0.5249	0.3135	0.055*	
C40	0.12157 (17)	0.38762 (17)	0.17216 (16)	0.0486 (4)	
H40	0.0571	0.4239	0.1560	0.058*	
N1	0.66387 (13)	−0.11021 (11)	0.28216 (11)	0.0369 (3)	
N2	0.70462 (14)	−0.05886 (11)	0.39516 (11)	0.0390 (3)	
N3	0.55049 (11)	0.25684 (10)	0.22935 (10)	0.0310 (2)	
O1	0.50184 (12)	0.37240 (10)	0.50186 (11)	0.0498 (3)	
O2	0.19856 (11)	0.09458 (9)	0.32322 (10)	0.0406 (2)	
O3	0.40291 (12)	0.00307 (9)	0.09023 (10)	0.0457 (3)	
S1	0.77092 (4)	0.31491 (4)	0.16018 (4)	0.04985 (13)	

### Atomic displacement parameters (Å^2^)

**Table:**

	*U*^11^	*U*^22^	*U*^33^	*U*^12^	*U*^13^	*U*^23^
C1	0.152 (2)	0.0546 (12)	0.0414 (10)	0.0537 (14)	−0.0089 (12)	0.0153 (9)
C2	0.211 (4)	0.0627 (15)	0.0592 (14)	0.080 (2)	−0.0104 (18)	0.0172 (12)
C3	0.126 (2)	0.0436 (11)	0.0538 (12)	0.0389 (13)	0.0121 (13)	0.0153 (9)
C4	0.0898 (15)	0.0462 (10)	0.0399 (9)	0.0178 (10)	0.0044 (9)	0.0140 (8)
C5	0.0823 (13)	0.0433 (9)	0.0416 (9)	0.0211 (9)	−0.0033 (9)	0.0183 (8)
C6	0.0496 (8)	0.0318 (7)	0.0376 (8)	0.0125 (6)	0.0057 (6)	0.0180 (6)
C7	0.0498 (8)	0.0337 (7)	0.0358 (7)	0.0139 (6)	0.0003 (6)	0.0190 (6)
C8	0.0368 (7)	0.0312 (7)	0.0355 (7)	0.0088 (5)	0.0015 (6)	0.0182 (6)
C9	0.0383 (7)	0.0330 (7)	0.0355 (7)	0.0075 (6)	0.0022 (6)	0.0187 (6)
C10	0.0471 (8)	0.0343 (7)	0.0334 (7)	0.0041 (6)	0.0015 (6)	0.0190 (6)
C11	0.0570 (10)	0.0475 (10)	0.0412 (9)	0.0133 (8)	0.0085 (7)	0.0208 (8)
C12	0.0896 (16)	0.0537 (12)	0.0460 (11)	0.0201 (11)	0.0176 (10)	0.0148 (9)
C13	0.1030 (19)	0.0669 (14)	0.0358 (10)	0.0085 (13)	−0.0045 (11)	0.0123 (9)
C14	0.0740 (14)	0.0706 (14)	0.0471 (11)	0.0046 (11)	−0.0158 (10)	0.0260 (10)
C15	0.0543 (10)	0.0492 (10)	0.0428 (9)	0.0081 (8)	−0.0027 (7)	0.0235 (8)
C16	0.0341 (6)	0.0274 (6)	0.0327 (7)	0.0073 (5)	−0.0009 (5)	0.0141 (5)
C17	0.0321 (6)	0.0286 (7)	0.0363 (7)	0.0086 (5)	0.0020 (5)	0.0158 (6)
C18	0.0334 (7)	0.0420 (8)	0.0537 (9)	0.0075 (6)	0.0011 (6)	0.0259 (8)
C19	0.0414 (8)	0.0434 (8)	0.0426 (8)	0.0094 (6)	0.0050 (6)	0.0266 (7)
C20	0.0321 (6)	0.0250 (6)	0.0287 (6)	0.0057 (5)	−0.0002 (5)	0.0110 (5)
C21	0.0325 (6)	0.0257 (6)	0.0277 (6)	0.0050 (5)	0.0006 (5)	0.0112 (5)
C22	0.0374 (7)	0.0303 (7)	0.0304 (7)	0.0063 (5)	0.0002 (5)	0.0122 (6)
C23	0.0396 (7)	0.0396 (8)	0.0316 (7)	0.0106 (6)	0.0044 (6)	0.0157 (6)
C24	0.0504 (9)	0.0471 (9)	0.0378 (8)	0.0150 (7)	0.0077 (7)	0.0134 (7)
C25	0.0575 (11)	0.0624 (12)	0.0496 (10)	0.0270 (9)	0.0185 (8)	0.0237 (9)
C26	0.0447 (9)	0.0748 (14)	0.0627 (12)	0.0223 (9)	0.0165 (9)	0.0381 (11)
C27	0.0387 (8)	0.0585 (11)	0.0556 (10)	0.0057 (7)	0.0047 (7)	0.0308 (9)
C28	0.0403 (7)	0.0414 (8)	0.0355 (7)	0.0080 (6)	0.0046 (6)	0.0208 (6)
C29	0.0379 (7)	0.0303 (7)	0.0393 (8)	0.0032 (6)	0.0016 (6)	0.0173 (6)
C30	0.0372 (7)	0.0302 (7)	0.0298 (7)	0.0057 (5)	0.0026 (5)	0.0109 (5)
C31	0.0335 (7)	0.0392 (8)	0.0300 (7)	0.0045 (6)	0.0007 (5)	0.0128 (6)
C32	0.0423 (8)	0.0525 (10)	0.0334 (8)	0.0025 (7)	−0.0013 (6)	0.0089 (7)
C33	0.0423 (9)	0.0805 (14)	0.0376 (9)	0.0048 (9)	−0.0103 (7)	0.0197 (9)
C34	0.0414 (9)	0.0811 (14)	0.0444 (9)	0.0182 (9)	−0.0022 (7)	0.0308 (10)
C35	0.0338 (7)	0.0574 (10)	0.0401 (8)	0.0129 (7)	0.0037 (6)	0.0260 (8)
C36	0.0306 (6)	0.0391 (8)	0.0311 (7)	0.0068 (5)	0.0029 (5)	0.0171 (6)
C37	0.0326 (6)	0.0304 (7)	0.0314 (7)	0.0078 (5)	0.0021 (5)	0.0147 (5)
C38	0.0421 (8)	0.0317 (7)	0.0394 (8)	0.0087 (6)	0.0014 (6)	0.0146 (6)
C39	0.0515 (9)	0.0387 (8)	0.0526 (10)	0.0199 (7)	0.0111 (8)	0.0224 (7)
C40	0.0441 (8)	0.0592 (11)	0.0570 (10)	0.0251 (8)	0.0100 (8)	0.0345 (9)
N1	0.0489 (7)	0.0314 (6)	0.0330 (6)	0.0123 (5)	0.0004 (5)	0.0162 (5)
N2	0.0503 (7)	0.0354 (7)	0.0342 (6)	0.0102 (6)	−0.0011 (5)	0.0184 (5)
N3	0.0308 (5)	0.0304 (6)	0.0353 (6)	0.0070 (4)	0.0014 (5)	0.0181 (5)
O1	0.0443 (6)	0.0350 (6)	0.0480 (7)	0.0035 (5)	−0.0053 (5)	0.0022 (5)
O2	0.0367 (5)	0.0355 (6)	0.0428 (6)	0.0005 (4)	0.0004 (4)	0.0145 (5)
O3	0.0548 (7)	0.0309 (5)	0.0429 (6)	0.0133 (5)	0.0006 (5)	0.0086 (5)
S1	0.0412 (2)	0.0578 (3)	0.0645 (3)	0.00799 (19)	0.01113 (19)	0.0404 (2)

### Geometric parameters (Å, º)

**Table:**

C1—C6	1.360 (2)	C20—N3	1.4561 (17)
C1—C2	1.382 (3)	C20—C37	1.5199 (18)
C1—H1	0.9300	C20—C30	1.5762 (19)
C2—C3	1.359 (3)	C20—C21	1.5871 (18)
C2—H2	0.9300	C21—C29	1.5229 (19)
C3—C4	1.352 (3)	C21—C22	1.5273 (19)
C3—H3	0.9300	C22—O1	1.2109 (18)
C4—C5	1.373 (3)	C22—C23	1.472 (2)
C4—H4	0.9300	C23—C28	1.392 (2)
C5—C6	1.371 (2)	C23—C24	1.393 (2)
C5—H5	0.9300	C24—C25	1.375 (3)
C6—N1	1.4171 (19)	C24—H24	0.9300
C7—N1	1.3522 (17)	C25—C26	1.383 (3)
C7—C8	1.366 (2)	C25—H25	0.9300
C7—H7	0.9300	C26—C27	1.369 (3)
C8—C9	1.4207 (19)	C26—H26	0.9300
C8—C16	1.5031 (18)	C27—C28	1.387 (2)
C9—N2	1.3337 (19)	C27—H27	0.9300
C9—C10	1.471 (2)	C28—O2	1.3619 (19)
C10—C11	1.385 (2)	C29—O2	1.4367 (18)
C10—C15	1.387 (2)	C29—H29A	0.9700
C11—C12	1.381 (3)	C29—H29B	0.9700
C11—H11	0.9300	C30—O3	1.2080 (17)
C12—C13	1.365 (3)	C30—C31	1.473 (2)
C12—H12	0.9300	C31—C32	1.370 (2)
C13—C14	1.379 (3)	C31—C36	1.398 (2)
C13—H13	0.9300	C32—C33	1.407 (3)
C14—C15	1.378 (3)	C32—H32	0.9300
C14—H14	0.9300	C33—C34	1.365 (3)
C15—H15	0.9300	C33—H33	0.9300
C16—C17	1.5377 (19)	C34—C35	1.414 (2)
C16—C21	1.5744 (18)	C34—H34	0.9300
C16—H16	0.9800	C35—C36	1.403 (2)
C17—N3	1.4488 (16)	C35—C40	1.408 (3)
C17—C18	1.517 (2)	C36—C37	1.404 (2)
C17—H17	0.9800	C37—C38	1.362 (2)
C18—S1	1.8190 (17)	C38—C39	1.418 (2)
C18—H18A	0.9700	C38—H38	0.9300
C18—H18B	0.9700	C39—C40	1.362 (3)
C19—N3	1.4410 (19)	C39—H39	0.9300
C19—S1	1.8187 (16)	C40—H40	0.9300
C19—H19A	0.9700	N1—N2	1.3516 (18)
C19—H19B	0.9700		
			
C6—C1—C2	119.36 (19)	C29—C21—C22	106.27 (11)
C6—C1—H1	120.3	C29—C21—C16	113.43 (11)
C2—C1—H1	120.3	C22—C21—C16	110.40 (11)
C3—C2—C1	121.0 (2)	C29—C21—C20	114.27 (11)
C3—C2—H2	119.5	C22—C21—C20	107.39 (10)
C1—C2—H2	119.5	C16—C21—C20	104.98 (10)
C4—C3—C2	119.44 (19)	O1—C22—C23	122.22 (14)
C4—C3—H3	120.3	O1—C22—C21	122.30 (13)
C2—C3—H3	120.3	C23—C22—C21	115.47 (12)
C3—C4—C5	120.17 (19)	C28—C23—C24	118.87 (15)
C3—C4—H4	119.9	C28—C23—C22	120.37 (14)
C5—C4—H4	119.9	C24—C23—C22	120.62 (14)
C6—C5—C4	120.58 (16)	C25—C24—C23	120.63 (18)
C6—C5—H5	119.7	C25—C24—H24	119.7
C4—C5—H5	119.7	C23—C24—H24	119.7
C1—C6—C5	119.36 (16)	C24—C25—C26	119.41 (18)
C1—C6—N1	120.22 (15)	C24—C25—H25	120.3
C5—C6—N1	120.37 (14)	C26—C25—H25	120.3
N1—C7—C8	108.02 (13)	C27—C26—C25	121.22 (17)
N1—C7—H7	126.0	C27—C26—H26	119.4
C8—C7—H7	126.0	C25—C26—H26	119.4
C7—C8—C9	103.96 (12)	C26—C27—C28	119.37 (17)
C7—C8—C16	126.20 (13)	C26—C27—H27	120.3
C9—C8—C16	129.46 (13)	C28—C27—H27	120.3
N2—C9—C8	111.39 (13)	O2—C28—C27	117.43 (15)
N2—C9—C10	118.23 (13)	O2—C28—C23	122.08 (13)
C8—C9—C10	130.34 (13)	C27—C28—C23	120.48 (16)
C11—C10—C15	118.73 (16)	O2—C29—C21	111.51 (11)
C11—C10—C9	122.01 (15)	O2—C29—H29A	109.3
C15—C10—C9	119.25 (15)	C21—C29—H29A	109.3
C12—C11—C10	120.43 (18)	O2—C29—H29B	109.3
C12—C11—H11	119.8	C21—C29—H29B	109.3
C10—C11—H11	119.8	H29A—C29—H29B	108.0
C13—C12—C11	120.2 (2)	O3—C30—C31	127.91 (14)
C13—C12—H12	119.9	O3—C30—C20	124.41 (13)
C11—C12—H12	119.9	C31—C30—C20	107.62 (11)
C12—C13—C14	120.2 (2)	C32—C31—C36	120.07 (14)
C12—C13—H13	119.9	C32—C31—C30	132.19 (15)
C14—C13—H13	119.9	C36—C31—C30	107.71 (12)
C15—C14—C13	119.8 (2)	C31—C32—C33	118.07 (17)
C15—C14—H14	120.1	C31—C32—H32	121.0
C13—C14—H14	120.1	C33—C32—H32	121.0
C14—C15—C10	120.59 (19)	C34—C33—C32	121.84 (16)
C14—C15—H15	119.7	C34—C33—H33	119.1
C10—C15—H15	119.7	C32—C33—H33	119.1
C8—C16—C17	111.83 (11)	C33—C34—C35	121.61 (16)
C8—C16—C21	117.44 (11)	C33—C34—H34	119.2
C17—C16—C21	104.14 (10)	C35—C34—H34	119.2
C8—C16—H16	107.7	C36—C35—C40	116.42 (15)
C17—C16—H16	107.7	C36—C35—C34	115.40 (16)
C21—C16—H16	107.7	C40—C35—C34	128.12 (15)
N3—C17—C18	103.38 (11)	C31—C36—C35	123.00 (14)
N3—C17—C16	103.13 (11)	C31—C36—C37	113.46 (12)
C18—C17—C16	119.69 (12)	C35—C36—C37	123.41 (14)
N3—C17—H17	110.0	C38—C37—C36	118.75 (13)
C18—C17—H17	110.0	C38—C37—C20	131.74 (13)
C16—C17—H17	110.0	C36—C37—C20	109.06 (12)
C17—C18—S1	103.54 (10)	C37—C38—C39	118.62 (15)
C17—C18—H18A	111.1	C37—C38—H38	120.7
S1—C18—H18A	111.1	C39—C38—H38	120.7
C17—C18—H18B	111.1	C40—C39—C38	122.53 (15)
S1—C18—H18B	111.1	C40—C39—H39	118.7
H18A—C18—H18B	109.0	C38—C39—H39	118.7
N3—C19—S1	103.29 (10)	C39—C40—C35	120.16 (14)
N3—C19—H19A	111.1	C39—C40—H40	119.9
S1—C19—H19A	111.1	C35—C40—H40	119.9
N3—C19—H19B	111.1	N2—N1—C7	111.75 (12)
S1—C19—H19B	111.1	N2—N1—C6	120.28 (11)
H19A—C19—H19B	109.1	C7—N1—C6	127.95 (13)
N3—C20—C37	109.51 (11)	C9—N2—N1	104.88 (11)
N3—C20—C30	113.12 (11)	C19—N3—C17	109.17 (11)
C37—C20—C30	102.09 (11)	C19—N3—C20	121.01 (11)
N3—C20—C21	99.91 (10)	C17—N3—C20	108.75 (10)
C37—C20—C21	120.89 (11)	C28—O2—C29	114.31 (12)
C30—C20—C21	111.76 (10)	C19—S1—C18	93.45 (7)
			
C6—C1—C2—C3	0.8 (5)	C22—C23—C28—C27	−177.10 (14)
C1—C2—C3—C4	0.8 (5)	C22—C21—C29—O2	61.29 (14)
C2—C3—C4—C5	−1.2 (4)	C16—C21—C29—O2	−177.24 (11)
C3—C4—C5—C6	−0.1 (4)	C20—C21—C29—O2	−56.95 (16)
C2—C1—C6—C5	−2.1 (4)	N3—C20—C30—O3	62.38 (18)
C2—C1—C6—N1	−179.7 (3)	C37—C20—C30—O3	179.95 (14)
C4—C5—C6—C1	1.8 (3)	C21—C20—C30—O3	−49.46 (19)
C4—C5—C6—N1	179.35 (18)	N3—C20—C30—C31	−114.91 (12)
N1—C7—C8—C9	−0.38 (17)	C37—C20—C30—C31	2.65 (14)
N1—C7—C8—C16	173.16 (14)	C21—C20—C30—C31	133.25 (12)
C7—C8—C9—N2	0.50 (17)	O3—C30—C31—C32	−1.8 (3)
C16—C8—C9—N2	−172.75 (14)	C20—C30—C31—C32	175.39 (17)
C7—C8—C9—C10	178.08 (15)	O3—C30—C31—C36	−179.50 (15)
C16—C8—C9—C10	4.8 (3)	C20—C30—C31—C36	−2.33 (16)
N2—C9—C10—C11	−141.17 (16)	C36—C31—C32—C33	0.2 (2)
C8—C9—C10—C11	41.4 (2)	C30—C31—C32—C33	−177.25 (17)
N2—C9—C10—C15	37.3 (2)	C31—C32—C33—C34	0.1 (3)
C8—C9—C10—C15	−140.12 (17)	C32—C33—C34—C35	0.2 (3)
C15—C10—C11—C12	0.6 (3)	C33—C34—C35—C36	−0.8 (3)
C9—C10—C11—C12	179.15 (17)	C33—C34—C35—C40	176.27 (18)
C10—C11—C12—C13	−0.5 (3)	C32—C31—C36—C35	−0.9 (2)
C11—C12—C13—C14	0.5 (4)	C30—C31—C36—C35	177.11 (14)
C12—C13—C14—C15	−0.5 (4)	C32—C31—C36—C37	−177.03 (14)
C13—C14—C15—C10	0.7 (3)	C30—C31—C36—C37	1.02 (17)
C11—C10—C15—C14	−0.7 (3)	C40—C35—C36—C31	−176.26 (15)
C9—C10—C15—C14	−179.25 (17)	C34—C35—C36—C31	1.2 (2)
C7—C8—C16—C17	−40.1 (2)	C40—C35—C36—C37	−0.6 (2)
C9—C8—C16—C17	131.79 (16)	C34—C35—C36—C37	176.91 (15)
C7—C8—C16—C21	80.22 (19)	C31—C36—C37—C38	173.97 (13)
C9—C8—C16—C21	−107.91 (17)	C35—C36—C37—C38	−2.1 (2)
C8—C16—C17—N3	151.11 (11)	C31—C36—C37—C20	0.79 (17)
C21—C16—C17—N3	23.32 (13)	C35—C36—C37—C20	−175.29 (13)
C8—C16—C17—C18	−94.90 (15)	N3—C20—C37—C38	−53.9 (2)
C21—C16—C17—C18	137.31 (12)	C30—C20—C37—C38	−174.06 (15)
N3—C17—C18—S1	−41.84 (13)	C21—C20—C37—C38	61.2 (2)
C16—C17—C18—S1	−155.70 (10)	N3—C20—C37—C36	118.04 (12)
C8—C16—C21—C29	2.85 (17)	C30—C20—C37—C36	−2.08 (14)
C17—C16—C21—C29	127.10 (12)	C21—C20—C37—C36	−126.82 (13)
C8—C16—C21—C22	121.98 (13)	C36—C37—C38—C39	3.4 (2)
C17—C16—C21—C22	−113.77 (12)	C20—C37—C38—C39	174.72 (14)
C8—C16—C21—C20	−122.58 (13)	C37—C38—C39—C40	−2.2 (3)
C17—C16—C21—C20	1.67 (13)	C38—C39—C40—C35	−0.5 (3)
N3—C20—C21—C29	−150.53 (11)	C36—C35—C40—C39	1.8 (2)
C37—C20—C21—C29	89.50 (15)	C34—C35—C40—C39	−175.25 (18)
C30—C20—C21—C29	−30.60 (15)	C8—C7—N1—N2	0.16 (18)
N3—C20—C21—C22	91.87 (12)	C8—C7—N1—C6	−177.99 (15)
C37—C20—C21—C22	−28.09 (16)	C1—C6—N1—N2	−4.8 (3)
C30—C20—C21—C22	−148.19 (11)	C5—C6—N1—N2	177.67 (16)
N3—C20—C21—C16	−25.63 (12)	C1—C6—N1—C7	173.2 (2)
C37—C20—C21—C16	−145.60 (12)	C5—C6—N1—C7	−4.3 (3)
C30—C20—C21—C16	94.30 (12)	C8—C9—N2—N1	−0.41 (17)
C29—C21—C22—O1	144.48 (15)	C10—C9—N2—N1	−178.31 (13)
C16—C21—C22—O1	21.09 (19)	C7—N1—N2—C9	0.16 (17)
C20—C21—C22—O1	−92.82 (16)	C6—N1—N2—C9	178.47 (13)
C29—C21—C22—C23	−37.07 (15)	S1—C19—N3—C17	−42.78 (13)
C16—C21—C22—C23	−160.46 (12)	S1—C19—N3—C20	−170.07 (10)
C20—C21—C22—C23	85.62 (14)	C18—C17—N3—C19	57.10 (15)
O1—C22—C23—C28	−173.95 (15)	C16—C17—N3—C19	−177.57 (12)
C21—C22—C23—C28	7.6 (2)	C18—C17—N3—C20	−168.95 (12)
O1—C22—C23—C24	10.3 (2)	C16—C17—N3—C20	−43.62 (14)
C21—C22—C23—C24	−168.12 (14)	C37—C20—N3—C19	−61.20 (16)
C28—C23—C24—C25	0.3 (3)	C30—C20—N3—C19	51.93 (16)
C22—C23—C24—C25	176.08 (16)	C21—C20—N3—C19	170.87 (12)
C23—C24—C25—C26	0.7 (3)	C37—C20—N3—C17	171.32 (11)
C24—C25—C26—C27	−0.8 (3)	C30—C20—N3—C17	−75.55 (14)
C25—C26—C27—C28	−0.2 (3)	C21—C20—N3—C17	43.38 (13)
C26—C27—C28—O2	−177.88 (15)	C27—C28—O2—C29	−158.75 (14)
C26—C27—C28—C23	1.3 (3)	C23—C28—O2—C29	22.10 (19)
C24—C23—C28—O2	177.82 (14)	C21—C29—O2—C28	−55.40 (16)
C22—C23—C28—O2	2.0 (2)	N3—C19—S1—C18	13.50 (11)
C24—C23—C28—C27	−1.3 (2)	C17—C18—S1—C19	16.32 (12)

### Hydrogen-bond geometry (Å, º)

Cg is the centroid of the C23–C28 benzene ring.

**Table:**

*D*—H···*A*	*D*—H	H···*A*	*D*···*A*	*D*—H···*A*
C7—H7···O3	0.93	2.47	3.207 (2)	136
C29—H29*A*···O3	0.97	2.45	3.074 (2)	122
C17—H17···O3	0.98	2.52	3.091 (2)	117
C38—H38···O1^i^	0.93	2.40	3.226 (2)	148
C1—H1···*Cg*^ii^	0.93	2.94	3.713 (3)	142

Symmetry codes: (i) −*x*+1, −*y*+1, −*z*+1; (ii) −*x*+1, −*y*, −*z*+1.
